# Is everyone invited to the discussion table? A bibliometric analysis COVID-19-related mental health literature

**DOI:** 10.1017/gmh.2022.37

**Published:** 2022-07-29

**Authors:** Nadir Yalcin, Izgi Bayraktar, Erdem Karabulut, Renato de Filippis, Florence Jaguga, Ruta Karaliuniene, Sachin Nagendrappa, Camille Noël, Margaret Isioma Ojeahere, Dorottya Ori, Ramdas Ransing, Fahimeh Saeed, Mohammadreza Shalbafan, Sheikh Shoib, Irfan Ullah, Ramyadarshni Vadivel, Bita Vahdani, Rodrigo Ramalho

**Affiliations:** 1 Department of Clinical Pharmacy, Faculty of Pharmacy, Hacettepe University, Ankara, Turkey; 2 Department of Biostatistics, Faculty of Medicine, Hacettepe University, Ankara, Turkey; 3 Psychiatry Unit, Department of Health Sciences, University Magna Graecia of Catanzaro, Viale Europa, Catanzaro, Italy; 4 Department of Mental Health, Moi Teaching & Referral Hospital, Eldoret, Kenya; 5 Clinic for Psychiatry and Psychotherapy, Elblandklinikum Radebeul, Academic Hospital Technical University Dresden, Dresden, Germany; 6 National Institute of Mental Health and Neurosciences, Bengaluru, India; 7 Child Psychiatry Department, Centre Hospitalier Universitaire Saint-Pierre, Université Libre de Bruxelles, Brussels, Belgium; 8 Child & Adolescent Psychiatry Hospital La Petite Maison ACIS, Chastre, Belgium; 9 Department of Psychiatry, Jos University Teaching Hospital, Jos, Plateau State, Nigeria; 10 Institute of Behavioural Sciences, Semmelweis University, Budapest, Hungary; 11 Heim Pal National Pediatric Institute, Department of Mental Health, Budapest, Hungary; 12 Department of Psychiatry, BKL Walawalkar Rural Medical College, Ratnagiri, Maharashtra, India; 13 Department of Psychiatry, Psychosis Research Center, University of Social Welfare and Rehabilitation Sciences, Tehran, Iran; 14 Mental Health Research Center, Psychosocial Health Research Institute (PHRI) and Department of Psychiatry, School of Medicine, Iran University of Medical Sciences, Tehran, Iran; 15 Department of Psychiatry, JawaharLal Nehru Memorial Hospital Rainawari, Kashmir, India; 16 Kabir Medical College, Gandhara University, Peshawar, Pakistan; 17 Community Mental Health and Addictions, Waikato District Health Board, Hamilton, New Zealand; 18 Department of Psychology, University of Tehran, Tehran, Iran; 19 Department of Social and Community Health, School of Population Health, The University of Auckland, Auckland, New Zealand

**Keywords:** Bibliometrics, global health, mental health, publishing, PubMed

## Abstract

**Background:**

The COVID-19 pandemic has captured the mental health discussion worldwide. Examining countries' representation in this discussion could prove instrumental in identifying potential gaps in terms of ensuring a truly global conversation in times of global crisis.

**Methods:**

We collected mental health and COVID-19-related journal articles published in PubMed in 2020. We focused on the corresponding authors' countries of affiliation to explore countries' representation. We also examined these articles' academic impact and correlations with their corresponding authors' countries of affiliation. Additional journals and countries' indicators were collected from the Web of Science and World Bank websites, respectively. Data were analyzed using the IBM SPSS Statistics and the VOSviewer software.

**Results:**

In total, 3492 publications were analyzed. Based on the corresponding author, high-income countries produced 61.9% of these publications. Corresponding authors from Africa, Latin America and the Caribbean, and the Middle East combined accounted for 11.8% of the publications. Europe hosted corresponding authors with the most publications and citations, and corresponding authors from North America had the largest mean journal impact factor.

**Conclusions:**

The global scientific discussion during the COVID-19 pandemic saw an increased contribution of academics from developing countries. However, authors from high-income countries have continued to shape this discussion. It is imperative to ensure the active participation of low- and middle-income countries in setting up the global mental health research agenda, particularly in situations of global crisis, such as the ongoing pandemic.

## Introduction

The COVID-19 pandemic has captured the global scientific conversation in an attempt to understand the harmful effects of the ongoing pandemic and the public health measures taken to control it (Kambhampati *et al*., 2020). As early as August 2020, the number of publications related to mental health-related concerns from around the world had already surpassed the number of all mental health-related publications regarding other epidemic outbreaks such as the West Africa Ebola and H1N1 (Maalouf *et al*., 2021). However, certain countries produced the significant majority of this global literature. In their analysis of COVID-19 publications in psychology-related Web of Science (WoS) categories, Ho *et al*. reported the United States (US) to be the most productive country in terms of investigations on the psychological impact of the pandemic (Ho *et al*., 2021*a*, 2021*b*). Similarly, Akintunde *et al*. (2021) and Chen *et al*. (2021) examined countries' contributions to international collaborations in mental health-related publications and found the US and China to be the two most productive countries during this global crisis.

There has also been a predominance of key journals in the scientific literature published during the COVID-19 pandemic. By April 2020, two very influential journals, *The British Medical Journal* and *The Lancet*, shared the highest number of editorial material, material meant to capture the opinion of its authors and guide publications foci (Ho *et al*., 2021*a*, 2021*b*). A bibliometric analysis of the top 50 cited papers as of May 2020 found that over 50% of the identified articles were published in only three journals, *The Lancet*, *The New England Journal of Medicine*, and the *Journal of American Medical Association* (ElHawary *et al*., 2020). The influential role of a few key journals is not a new trend either. In 2009, Kieling *et al*. stated that the vast majority of indexed psychiatric journals were from high-income countries, with less than 5% originating from middle-income countries and none from low-income countries (Kieling *et al*., 2009).

The predominance of high-income countries in the mental health literature is not a new trend (Saxena and Sharan, 2004). In an assessment of child and adolescent psychiatric/psychological academic output, Albayrak *et al*. found a significant difference between high- and low-income countries in favor of high-income countries (Albayrak *et al*., 2012). Zhang *et al*. reported in 2017 that most psychiatry publications originated in high-income countries (Zhang *et al*., 2017). They also noted that about 1% of these publications came from low- and lower-middle-income countries, with the US having both the most citations and second-highest mean number of citations.

Nevertheless, the pandemic has represented an undeniable global affair. So, to better understand countries' representation and potential gaps the scientific community may be facing to ensure a truly global discussion, it could prove instrumental to examine the scientific outputs during the COVID-19 pandemic. For this purpose, we conducted a bibliometric analysis of mental health and COVID-19-related journal articles published in 2020 to better understand countries' contributions to the global mental health discussion during the first year of the pandemic.

The present study paid particular attention to the corresponding authors' countries of affiliation. Previous studies of this literature have homogenized the contributions of all authors in international collaborations. However, the role of the corresponding author is associated with a leadership role and the shaping of the study and the article (Chinchilla-Rodríguez *et al*., 2019; He *et al*., 2020). Previous studies have found low- and middle-income countries to have limited presence as corresponding authors in health-related literature (González-Alcaide *et al*., 2017; Fox *et al*., 2018). So, focusing on the corresponding author may add further detail and insights about countries' representation in the global COVID-19-related mental health literature. The present study also explored differences in the impact of these articles in terms of citations and journal of publication, e.g. journals' impact factor and ranking, and their correlation with the corresponding authors' countries of affiliation.

## Method

### Data collection

**Fig. 1.**
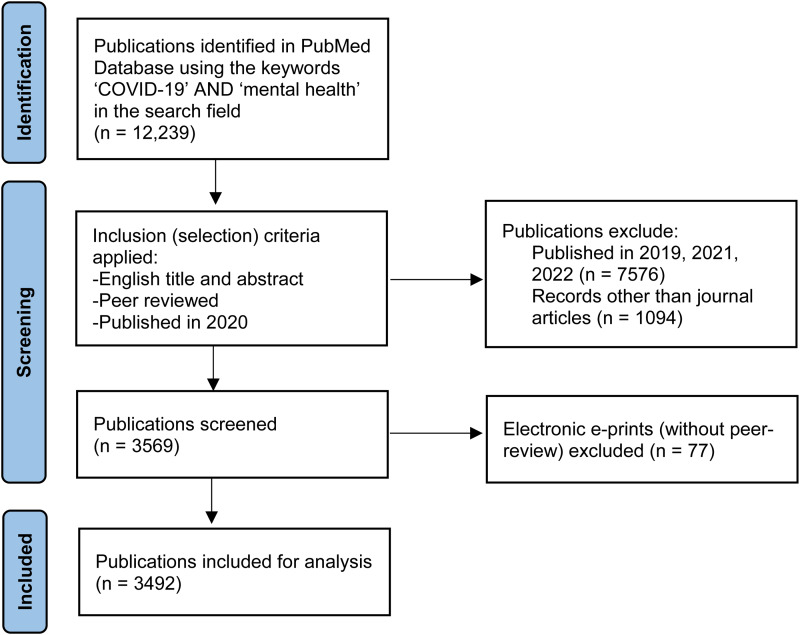
Flow diagram of data identification, screening, and inclusion.

In July 2021, we conducted a bibliometric analysis of mental health and COVID-19-related journal articles published in the PubMed Database in 2020. We carried out a comprehensive search in the PubMed Database using the keywords ‘COVID-19’ and ‘mental health’ in the search field. PubMed was selected as it is a free database, with no registration required, to which all authors had access, and fast in terms of publication speed. This first search led to 12 239 documents (Fig. 1). We included all ‘journal article’ document types with an English abstract that were peer-reviewed and published in 2020 and excluded other document types, documents with abstracts in other languages, and non-peer-reviewed publications, leading to a total of 3569 publications. Finally, 77 articles were excluded because they were published as electronic eprints without peer-review (medRxiv, F1000Research, etc.). A final total of 3492 articles were included in the analysis. The full record of the included publications was downloaded in CSV format, and the countries of affiliation of all listed authors in articles with authors from more than one country were used to explore inter-country collaborations. The following data were manually collected from each publication: the corresponding author's country of affiliation, the journal in which the article was published, and the number of citations listed in the PubMed database at the time of data collection. When there was more than one corresponding author, the senior (last author) was the one included for analysis, and if the corresponding author had more than one affiliation, only the first one was included for analysis. Further information collected from the top 10 cited articles included title, aim of the study, study period, study type, and number of participants.

Having identified in PubMed the corresponding authors' country of affiliation for each publication, we then collected the country classification by income level from the World Bank website. Having identified in PubMed the journals in which the included articles were published, we also then collected the following information from the WoS Journal Citation Reports™ 2020 (if available for the journal): (i) type of journal in which the article indexed in the WoS Core Collection was published, i.e. Science Citation Index Expanded (SCIE), Social Sciences Citation Index (SSCI), or Emerging Sources Citation Index (ESCI), (ii) whether the journal was Open Access, (iii) the Journal Impact Factor (JIF), and (iv) the Quartile Category (Q) according to the JIF.

### Data analysis

Continuous variables were defined as the mean (standard deviation, s.d.) and were compared using *t* tests. The normality of continuous data was tested using a Shapiro–Wilk test. Categorical variables were defined as percentages and were compared using the χ^2^ test. Correlations between quantitative variables were examined with Pearson or Spearman correlation coefficient. Chi-square analysis was used for the relationships between qualitative variables. For the comparisons between groups, appropriate tests such as the independent samples *t* test, Mann–Whitney *U* test, and one-way analysis of variance were performed considering the number of groups and the distribution of variables. Univariate analysis was carried out in *IBM SPSS Statistics Version 23* software. For all tests, *p* < 0.05 was considered statistically significant. In addition, we used the *IBM SPSS Statistics Version 23* software for creating a world map in terms of distribution of the mean number of articles, citations, *Q* rank, and JIF per country, according to the corresponding author.

To assess inter-country collaborations, we used the *VOSviewer* software (version 1.6.17) to create a graph-based map (Van Eck and Waltman, 2010). We considered the country of affiliation of all listed authors in articles with authors from more than one country. In the developed graph, each country is indicated as a circle. A country was included in the graph if it hosted authors in at least three articles. The size of the circle indicates the number of times an article had an author from that country. That is, the larger the circle, the greater the number of authors were from that country. The lines between the circles indicate collaboration between countries, and the thickness increases as the number of collaborations between countries increases, i.e. the thicker the line, the larger the number of collaborations.

## Results

Table 1 presents the characteristics of the included articles. The corresponding author was from a high-income country in 61.9% of all publications. Less than 1% of the total number of publications had a corresponding author from a low-income country. Sub-Saharan Africa hosted a corresponding author in less than 2% of the total number of publications, 4.1% for Latin America and the Caribbean, and 6.3% for the Middle East and North Africa. These regions combined hosted a corresponding author in 11.8% of all publications.

**Table 1.** Description of the included articles

Variables	*n* (%)
Journal Indexes (*N* = 3492)	
SCIE	1487 (42.6%)
SSCI	1404 (40.2%)
ESCI	601 (17.2%)
Open Access (*N* = 3492)	
Yes	1439 (41.2%)
No	2053 (58.8%)
Quartile category (*N* = 2891)	
Q1	1096 (37.9%)
Q2	1112 (38.5%)
Q3	352 (12.2%)
Q4	331 (11.4%)
Country income level (*N* = 3492)^a^	
Low income	15 (0.4%)
Lower-middle income	559 (16.0%)
Upper-middle income	756 (21.6%)
High income	2162 (61.9%)
Regions (*N* = 3492)^a^	
East Asia and Pacific	810 (23.2%)
Europe and Central Asia	1210 (34.7%)
Latin America and Caribbean	144 (4.1%)
Middle East and North Africa	219 (6.3%)
North America	646 (18.5%)
South Asia	414 (11.9%)
Sub-Saharan Africa	49 (1.4%)

SCIE, Science Citation Index Expanded™; SSCI, Social Sciences Citation Index™; ESCI, Emerging Sources Citation Index™.

a The distribution by country classification by income level and region was done according to the corresponding authors' country of affiliation.

Table 2 offers a more detailed description of the number of articles and citations distributed by corresponding authors' countries of affiliation. In Europe and Central Asia, the distribution of publications per country is relatively homogeneous, except for Italy and the United Kingdom (UK), which hosted a corresponding author for 6.2% and 8.1% of the total number of publications, respectively. Similarly, East Asia and the Pacific have a relatively homogenous distribution of publications per country, with the exceptions of China, hosting a corresponding author in 13% of the total number of publications, and Australia, in 4.4% of the total. Two countries from North America, the US and Canada, hosted corresponding authors in 12.7% and 5.8% of the total number, adding up to 18.5%. Sub-Saharan Africa, Latin America and the Caribbean, and the Middle East and North Africa had a relatively homogenous distribution of publications per country. Online Supplementary Material 1 shows a world map indicating the mean number of publications per country based on the corresponding author.

**Table 2.** Distribution of the number (No) of articles and median No of citations by country and region, according to the corresponding author

Country	Region	No of articles (%)	Median No of citations (min–max)
Australia	East Asia and Pacific	155 (4.4%)	2 (0–177)
China	East Asia and Pacific	454 (13.0%)	4 (0–1569)
Hong Kong	East Asia and Pacific	9 (0.3%)	5 (0–12)
Indonesia	East Asia and Pacific	8 (0.2%)	1 (0–6)
Japan	East Asia and Pacific	43 (1.2%)	3 (0–79)
Korea	East Asia and Pacific	24 (0.7%)	1 (0–22)
Malaysia	East Asia and Pacific	21 (0.6%)	2 (0–22)
New Zealand	East Asia and Pacific	18 (0.5%)	0.5 (0–49)
Papua New Guinea	East Asia and Pacific	1 (0.0%)	4 (4–4)
Philippines	East Asia and Pacific	8 (0.2%)	7 (0–34)
Singapore	East Asia and Pacific	41 (1.2%)	2 (0–446)
Taiwan	East Asia and Pacific	12 (0.3%)	2 (0–43)
Thailand	East Asia and Pacific	7 (0.2%)	3 (0–15)
Vietnam	East Asia and Pacific	10 (0.3%)	3.5 (0–33)
Albania	Europe and Central Asia	1 (0.0%)	0 (0–0)
Austria	Europe and Central Asia	19 (0.5%)	5 (0–55)
Belarus	Europe and Central Asia	1 (0.0%)	1 (1–1)
Belgium	Europe and Central Asia	22 (0.6%)	3.5 (0–690)
Bosnia and Herzegovina	Europe and Central Asia	5 (0.1%)	1 (0–12)
Bulgaria	Europe and Central Asia	1 (0.0%)	4 (4–4)
Croatia	Europe and Central Asia	17 (0.5%)	2 (0–40)
Cyprus	Europe and Central Asia	3 (0.1%)	4 (1–39)
Czech Republic	Europe and Central Asia	7 (0.2%)	1 (0–3)
Denmark	Europe and Central Asia	12 (0.3%)	4 (0–214)
Finland	Europe and Central Asia	2 (0.1%)	1 (0–2)
France	Europe and Central Asia	52 (1.5%)	2 (0–54)
Georgia	Europe and Central Asia	1 (0.0%)	1 (1–1)
Germany	Europe and Central Asia	78 (2.2%)	3 (0–102)
Greece	Europe and Central Asia	27 (0.8%)	3 (0–44)
Hungary	Europe and Central Asia	6 (0.2%)	0 (0–48)
Iceland	Europe and Central Asia	2 (0.1%)	1.5 (0–3)
Ireland	Europe and Central Asia	61 (1.7%)	2 (0–73)
Italy	Europe and Central Asia	217 (6.2%)	3 (0–395)
Kazakhstan	Europe and Central Asia	2 (0.1%)	0.5 (0–1)
Lithuania	Europe and Central Asia	1 (0.0%)	3 (3–3)
Luxembourg	Europe and Central Asia	1 (0.0%)	0 (0–0)
Malta	Europe and Central Asia	2 (0.1%)	1.5 (1–2)
Netherlands	Europe and Central Asia	39 (1.1%)	4 (0–111)
North Macedonia	Europe and Central Asia	2 (0.1%)	1 (0–2)
Norway	Europe and Central Asia	15 (0.4%)	3 (0–49)
Poland	Europe and Central Asia	24 (0.7%)	4 (0–27)
Portugal	Europe and Central Asia	25 (0.7%)	2 (0–23)
Romania	Europe and Central Asia	7 (0.2%)	2 (0–4)
Russia	Europe and Central Asia	6 (0.2%)	0 (0–1)
Serbia	Europe and Central Asia	7 (0.2%)	0 (0–18)
Slovakia	Europe and Central Asia	2 (0.1%)	1 (0–2)
Slovenia	Europe and Central Asia	1 (0.0%)	4 (4–4)
Spain	Europe and Central Asia	137 (3.9%)	2 (0–155)
Sweden	Europe and Central Asia	25 (0.7%)	5 (0–53)
Switzerland	Europe and Central Asia	32 (0.9%)	2.5 (0–84)
Turkey	Europe and Central Asia	63 (1.8%)	2 (0–121)
Ukraine	Europe and Central Asia	3 (0.1%)	0 (0–0)
United Kingdom	Europe and Central Asia	284 (8.1%)	3 (0–800)
Argentina	Latin America and Caribbean	14 (0.4%)	2 (0–15)
Brazil	Latin America and Caribbean	81 (2.3%)	3 (0–115)
Chile	Latin America and Caribbean	6 (0.2%)	1.5 (0–5)
Colombia	Latin America and Caribbean	10 (0.3%)	0 (0–11)
Dominican Republic	Latin America and Caribbean	3 (0.1%)	0 (0–7)
Ecuador	Latin America and Caribbean	2 (0.1%)	2 (0–4)
Mexico	Latin America and Caribbean	10 (0.3%)	1 (0–6)
Panama	Latin America and Caribbean	2 (0.1%)	3.5 (3–4)
Paraguay	Latin America and Caribbean	2 (0.1%)	3.5 (3–4)
Peru	Latin America and Caribbean	15 (0.4%)	0 (0–47)
Trinidad and Tobago	Latin America and Caribbean	1 (0.0%)	109 (109–109)
Egypt	Middle East and North Africa	12 (0.3%)	1.5 (0–36)
Iran	Middle East and North Africa	72 (2.1%)	3 (0–269)
Iraq	Middle East and North Africa	3 (0.1%)	2 (1–56)
Israel	Middle East and North Africa	36 (1.0%)	2 (0–71)
Jordan	Middle East and North Africa	9 (0.3%)	2 (0–54)
Kingdom of Bahrain	Middle East and North Africa	1 (0.0%)	1 (1–1)
Lebanon	Middle East and North Africa	12 (0.3%)	3.5 (0–14)
Libya	Middle East and North Africa	3 (0.1%)	2 (0–12)
Morocco	Middle East and North Africa	5 (0.1%)	1 (0–12)
Oman	Middle East and North Africa	5 (0.1%)	3 (0–6)
Palestine	Middle East and North Africa	2 (0.1%)	1 (0–2)
Qatar	Middle East and North Africa	8 (0.2%)	1.5 (0–28)
Saudi Arabia	Middle East and North Africa	39 (1.1%)	2 (0–41)
Tunisia	Middle East and North Africa	4 (0.1%)	7 (3–14)
United Arab Emirates	Middle East and North Africa	9 (0.3%)	3 (0–28)
Canada	North America	203 (5.8%)	2 (0–116)
United States of America	North America	445 (12.7%)	2 (0–500)
Bangladesh	South Asia	34 (1.0%)	7 (0–70)
India	South Asia	300 (8.6%)	1 (0–505)
Nepal	South Asia	16 (0.5%)	1.5 (0–26)
Pakistan	South Asia	63 (1.8%)	2 (0–53)
Sri Lanka	South Asia	3 (0.1%)	4 (1–5)
Cameroon	Sub-Saharan Africa	3 (0.1%)	1 (0–1)
Ethiopia	Sub-Saharan Africa	10 (0.3%)	1 (0–5)
Ghana	Sub-Saharan Africa	1 (0.0%)	0 (0–0)
Kenya	Sub-Saharan Africa	3 (0.1%)	2 (0–3)
Mali	Sub-Saharan Africa	1 (0.0%)	1 (1–1)
Nigeria	Sub-Saharan Africa	12 (0.3%)	0.5 (0–16)
Rwanda	Sub-Saharan Africa	1 (0.0%)	0 (0–0)
South Africa	Sub-Saharan Africa	15 (0.4%)	3 (0–58)
Togo	Sub-Saharan Africa	1 (0.0%)	1 (1–1)
Uganda	Sub-Saharan Africa	2 (0.1%)	2 (1–3)
Zimbabwe	Sub-Saharan Africa	1 (0.0%)	3 (3–3)
**Total**		**3492 (100.0%)**	**2 (0–1569)**

Table 2 reports the median, minimum, and maximum number of citations per country. Countries with the maximum number of citations, based on corresponding authors, are China (*n* = 1569), UK (*n* = 800), Belgium (*n* = 690), India (*n* = 505), and the US (*n* = 500). Europe is the continent that hosted corresponding authors with more citations, followed by Asia and North America, with Africa as the continent with the lowest sum of citations. Online Supplementary Material 2 shows a world map indicating the mean number of citations by country, based on the corresponding author.

As shown in Table 1, most of the included articles were published in Q1 and Q2 journals, 37.9% and 38.5%, respectively. Also, the majority of included articles were indexed in SCIE and SSCI, 42.6% and 40.2%, respectively. Table 3 shows the distribution of Open Access and non-Open Access articles. Out of the total number of articles, 2053 (58.8%) were published Open Access [*v.* non-Open Access (*n* = 1439; 41.2%; *p* > 0.05)]. Most articles published in Q2, Q3, and Q4 were Open Access (*p* < 0.0001), unlike articles published in Q1 journals (*p* > 0.05). The mean number of citations in non-Open Access articles was 3 (0–800) [*v.* 2 (0–1569) in Open Access articles (*p* = 0.029)]. There was no statistically significant difference between the JIF in Open Access *v.* non-Open Access articles. Looking at the regions from where these articles originated, the majority of articles coming from Europe and Central Asia, South Asia, and East Asia and Pacific were Open Access (*p* < 0.05), unlike articles originating from Latin America and the Caribbean, Middle East and North Africa, and Sub-Saharan Africa (*p* > 0.05).

**Table 3.** Distribution of Open Access and non-Open Access articles

	OA	Non-OA	*p* value
Journal indexes (*N* = 3492)			
SCIE	866 (58.2%)	621 (41.8%)	>0.05
SSCI	796 (56.7%)	608 (43.3%)	0.002
ESCI	210 (34.9%)	391 (65.1%)	0.002
Quartile category (*N* = 2891)			
Q1	627 (57.2%)	469 (42.8%)	>0.05
Q2	694 (62.4%)	418 (37.6%)	<0.0001
Q3	173 (49.1%)	179 (50.9%)	<0.0001
Q4	168 (50.8%)	163 (49.2%)	<0.0001
Country income level (*N* = 3492)^a^			
Low income	12 (80.0%)	3 (20.0%)	>0.05
Lower-middle income	351 (62.8%)	208 (37.2%)	0.034
Upper-middle income	451 (59.7%)	305 (40.3%)	>0.05
High income	1239 (57.3%)	923 (42.7%)	0.034
Regions (*N* = 3492)^a^			
East Asia and Pacific	501 (61.9%)	309 (38.1%)	0.017
Europe and Central Asia	728 (60.2%)	482 (39.8%)	0.017
Latin America and Caribbean	83 (57.6%)	61 (42.4%)	>0.05
Middle East and North Africa	129 (58.9%)	90 (41.1%)	>0.05
North America	323 (50.0%)	323 (50.0%)	>0.05
South Asia	258 (62.3%)	156 (37.7%)	0.017
Sub-Saharan Africa	31 (63.3%)	18 (36.7%)	>0.05
Number of articles (*N* = 3492)	2053 (58.8%)	1439 (41.2%)	>0.05
Number of citations (*N* = 3492)	2 (0–1569)	3 (0–800)	0.029
JIF (*N* = 2891)	3.390 (0.110–52.320)	3.307 (0.030–79.321)	>0.05

SCIE, Science Citation Index Expanded™; SSCI, Social Sciences Citation Index™; ESCI, Emerging Sources Citation Index™.

a The distribution by country classification by income level and region was done according to the corresponding authors' country of affiliation

Online Supplementary Material 3 shows a world map indicating the mean number of *Q* rank per country, based on the corresponding author. Online Supplementary Material 4 shows a world map indicating the mean journal impact factor of published articles per country, based on the corresponding author. North America hosted corresponding authors who published articles in journals with the largest mean journal impact factor, followed by Europe and Australia. Africa hosted corresponding authors who published articles in journals with the lowest median journal impact factor. Online Supplementary Material 5 shows a graph indicating inter-country collaborations between all authors in the included articles. As can be seen in the graph, when considering co-authorship in international collaborations, the countries with the larger number of authors were the US and China. The US had the strongest international collaboration network (with 1020 total link strength), followed by the UK (966), Italy (692), and Spain (503).

Finally, online Supplementary Materials 6 and 7 present the top 10 cited publications among the total number of publications included in this study. Five of the top 10 cited papers had a corresponding author from China, with the other five publications having corresponding authors from India, Singapore, Canada, and two from the UK. All 10 papers were WoS indexed (SCIE or SSCI), and the journals in which they were published were ranked as Q1 or Q2. Most of these articles were focused on assessing the mental health situation, prevalence, or effects of the pandemic, as well as risk and protective factors, in the general population or special populations, such as healthcare workers or students.

## Discussion

The present bibliometrics analysis explored the worldwide scientific output regarding COVID-19 and mental health during 2020, as found in the PubMed database. This is the first study to examine the global scientific discussion on mental health during the pandemic to pay attention to the corresponding author. This focus provides a more detailed and insightful view of countries' representation in the global COVID-19-related mental health literature. Looking at the corresponding author's affiliation, the majority of the literature was led by authors from high-income countries, i.e. about 62% of these publications, with less than 1% being led by authors from low-income countries. Most publications led by an author from high-income countries were published in journals ranked in the top 25% (Q1 Journals). Europe was the region that hosted corresponding authors with the most publications and citations, and corresponding authors from North America had published articles in journals with the largest mean journal impact factor. Only about 12% of the total number of included publications were led by an author from Africa, the Middle East, or Latin America and the Caribbean combined.

Akintunde *et al*. also conducted a bibliometric overview of mental health-related publications during the pandemic, although they used the WoS database (Akintunde *et al*., 2021). Their study showed that about 71% of all publications originated from developing countries. This number, however, included countries listed in inter-country co-authorship collaborations, and it homogenized the contributions of all authors in each collaboration. Chen *et al*. also explored mental health-related publications from the WoS (Chen *et al*., 2021). Like the present study, they found the US and China to be the two most productive countries when assessing inter-country collaborations. According to Chen *et al*. these two countries accounted for almost half of all articles found in that database. A previous bibliometric analysis of depression and COVID-19-related research also showed that the US had the highest number of published papers (Al-Jabi, 2021). So, while there has been a growing contribution in terms of co-authorship from developing countries during the pandemic, the US has remained one of the most productive countries in terms of mental health-related scientific output during the pandemic.

To see more authors from developing countries collaborating in the global discussion and authors from high-income countries reaching out to these authors represents a step forward. While the US was one of the most productive countries in terms of hosting corresponding authors in the literature included in the present study, it was also the country with the strongest international collaboration network. But participating in international collaborations is sometimes a matter of necessity for authors from some countries, who otherwise may have little to no outputs (de Moya-Anegon *et al*., 2018). For example, a study in Brazil, an upper-middle-income country in Latin America, reported that their international collaborations had a better impact when a non-Brazilian was the corresponding author (Grácio *et al*., 2020). The US seems to be the only country that benefits from having the leading role in an international collaboration in terms of citations (Chinchilla-Rodríguez *et al*., 2019). So, while the pandemic saw more countries contributing to the global discussion, certain regions and countries may have continued to shape this discussion.

The present study also found that, unlike Latin America and the Caribbean, the Middle East, and Sub-Saharan and North Africa regions, Europe was one of the regions with a majority of Open Access publications. Open Access publications seem to have a citation advantage and further non-academic dissemination (Tennant *et al*., 2016; Hafeez *et al*., 2019), although the evidence in this area seems inconclusive (Langham-Putrow *et al*., 2021). Moreover, in the present study, non-Open Access publications had a greater mean number of citations than Open Access publications. A study conducted by Davis *et al*. (2008) found that Open Access publications did not show citation advantage; however, they tended to reach more readers than non-Open Access publications. This contributes, for example, to the diffusion of treatment research in mental health (Hardisty and Haaga, 2008). This suggests that Europe, having the possibility to reach a wider audience during this early period, Europe might have had a significant influence in shaping the global discussion during the first year of the pandemic.

Europe was also the continent that hosted corresponding authors with more citations, followed by Asia and North America. On the other hand, North America hosted corresponding authors who published articles in journals with the largest mean journal impact factor, followed by Europe. It has been suggested that early citations play a more significant role in the long-term scholarly impact of a publication than the JIF (Abramo *et al*., 2019; Ruan *et al*., 2020). This suggests that Europe may have a more lasting scholarly impact on the COVID-19 mental health-related literature. Nevertheless, the JIF continues to impact how publications are perceived and received by the academic community, as it is seen as a marker of quality (Cleary *et al*., 2013). So, it is likely that both Europe and North America will continue to exert a significant impact on shaping the discussion in this area. It is important to highlight that the present study found authors from Asia contributing substantially to the global discussion during the pandemic, as also reported by Akintunde *et al*. (2021) and Chen *et al*. (2021).

Still, researchers from low- and middle-income countries, particularly from the Global South, continue to face multiple barriers to publication (El Halabi *et al*., 2021). North-South collaborations or partnerships through funding, mentorship, and research support could assist in diminishing these barriers (Merritt *et al*., 2019; El Halabi *et al*., 2021). However, these collaborations run the risk of creating further disadvantages, mainly if existing power relations and inequitable access to resources contribute to the partners in the North setting up the research agenda (Franzen *et al*., 2017). Ideally, health research capacity strengthening in the Global South should empower local researchers to define local priorities systematically and create, apply, and share all generated solutions. This type of approach seems especially appropriate during global crises such as the ongoing pandemic, where differences between settings may hinder the transferability of studies when prompt action is required.

At the same time, national policies in developing countries should promote the contribution of local research centers and researchers to the national growth and development (GrAy, 2008). Policies that focus on rewarding individual achievement in international rankings force researchers to invest in a research agenda that resonates with international audiences rather than respond to national needs. It also encourages researchers from low- and middle-income countries to follow a foreign agenda rather than lead a local one to increase the chances of publishing in high-ranking journals and being widely cited. At the same time, these policies must include a mental health and wellbeing research agenda. This research agenda should not be set up separately from other social or economic policies but rather across them via multi-sector coalitions that include community-based organizations.

The present study is not without limitations. A unique database (PubMed) was used to obtain publications. Thus, publications included in other databases were not included in the analysis. Also, the present study only included articles published during the first year of the pandemic, but the trend reflected in the findings may have changed as the pandemic progressed. Future research in this area should aim to include other databases and expand the data collection period beyond the first year of the pandemic. In an attempt to be comprehensive, the broadness of the two keywords used in the study has very likely led to the inclusion of articles that, while mentioning mental health and COVID-19, were not solely focused on COVID-19-related mental health mental issues. Future research would also benefit from a further screening process to identify and include articles focused on mental health and COVID-19. Finally, most publications in PubMed are in English, and at the same time, we included only journal articles with English abstracts. This represents a limitation in a study that aims to explore the global production of academic outputs and a missed opportunity, considering the wide range of languages spoken by the research team.

## Conclusion

The COVID-19 pandemic, an undeniable global affair, has seen the world uniting to discuss how to best address its worldwide impact. The present study explored scientific publications during 2020 regarding mental health, paying attention to the corresponding author. Although there was an increased number of contributions from developing countries during the pandemic compared to previous years, high-income countries have continued to shape the global discussion. Moreover, the more significant scholarly impact of publications led by authors from high-income countries suggests they will continue to shape this conversation in the future when looking back at the pandemic, its impact, and the strategies used to confront it. It is vital to strengthen research capacity in low- and middle-income countries. But, most importantly, it is imperative to ensure their active participation in setting up the global mental health research agenda, particularly in a global crisis, such as the ongoing pandemic.

## Supplementary material

For supplementary material accompanying this paper visit https://doi.org/10.1017/gmh.2022.37. click here to view supplementary material
