# Supplementary material for: Is everyone invited to the discussion table? A bibliometric analysis COVID-19-related mental health literature
Source: Glob Ment Health (Camb). 2022 Jul 29;9:366–74. doi: 10.1017/gmh.2022.37 (PMC9379265; doi:10.1017/gmh.2022.37)
Supplement: Supplementary file 1 [file S2054425122000371sup.zip › S2054425122000371sup001.docx]

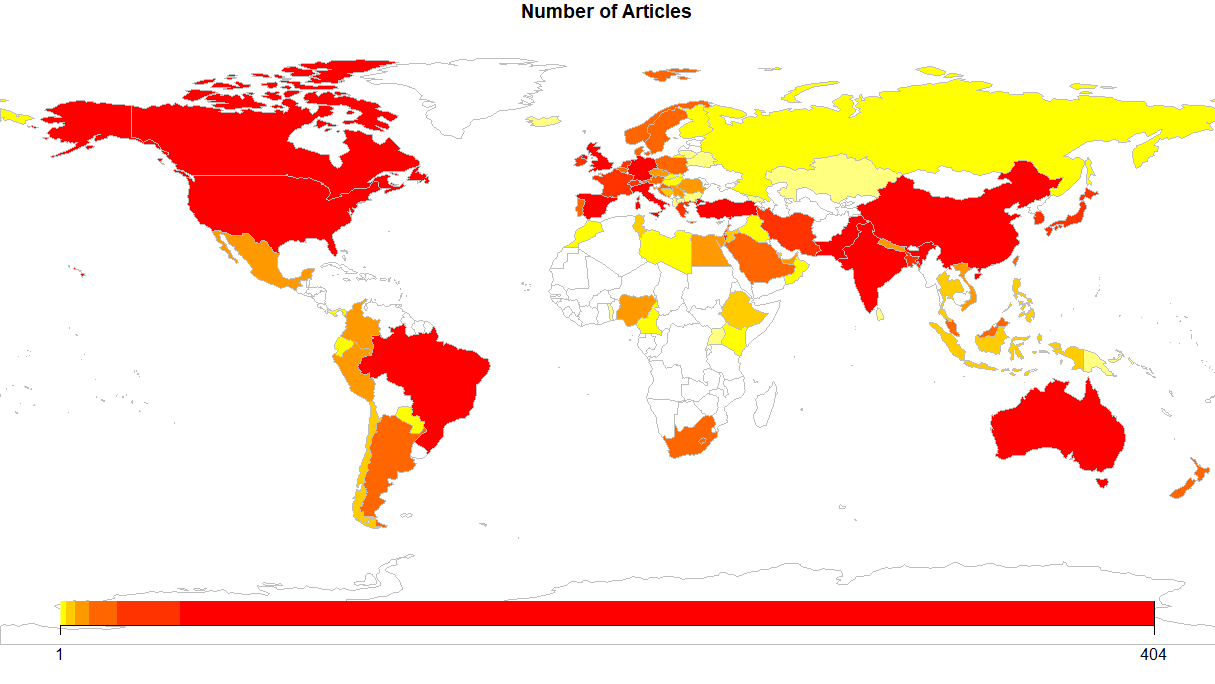
Supplementary Material 1. Mean number of articles per country based on the corresponding author


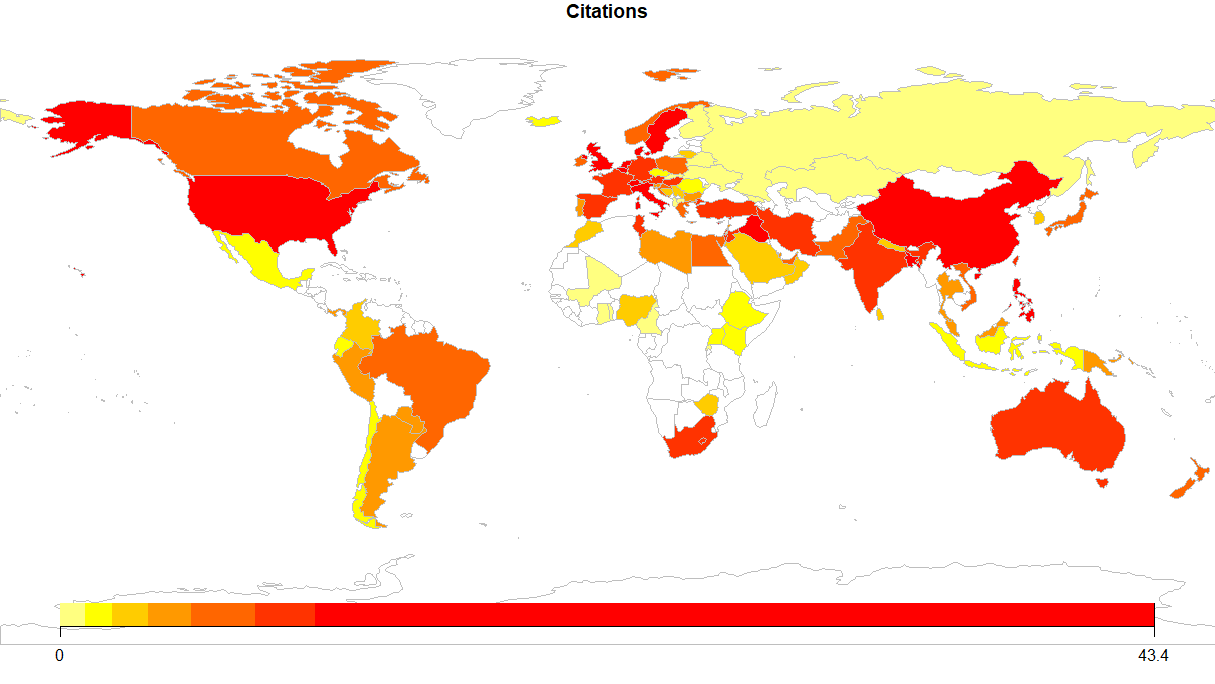


Supplementary Material 2. Mean number of citations per country based on the corresponding author


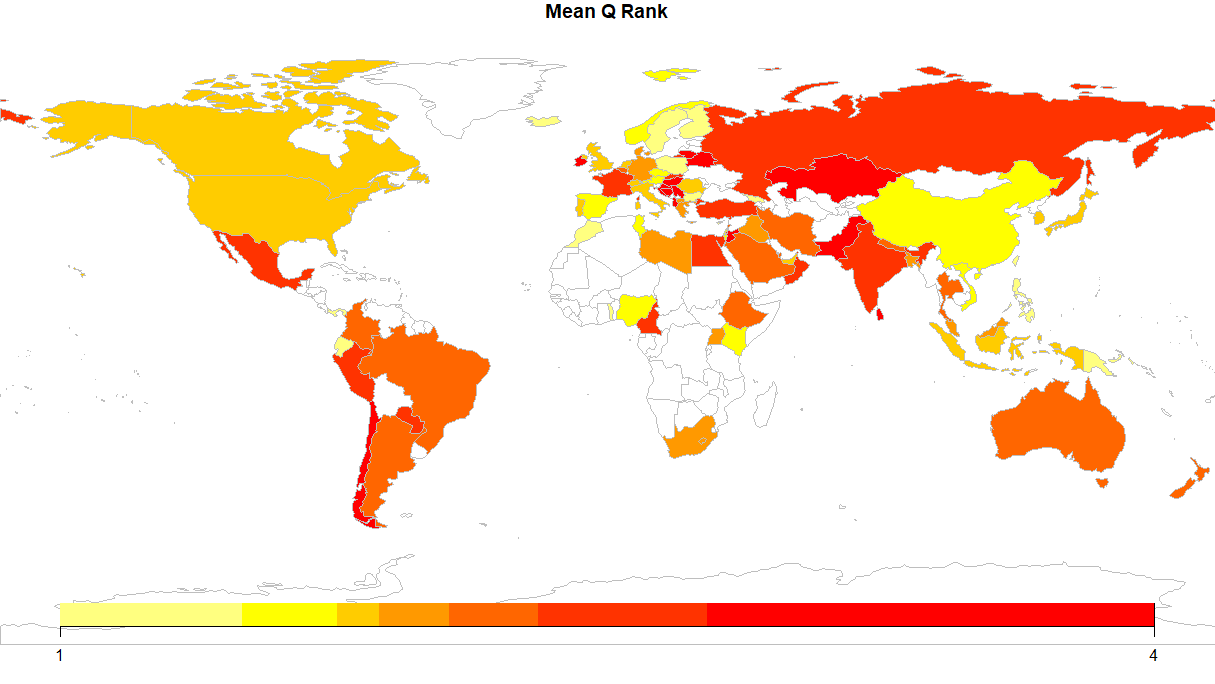


Supplementary Material 3. Mean number of Q Rank per country based on the corresponding author


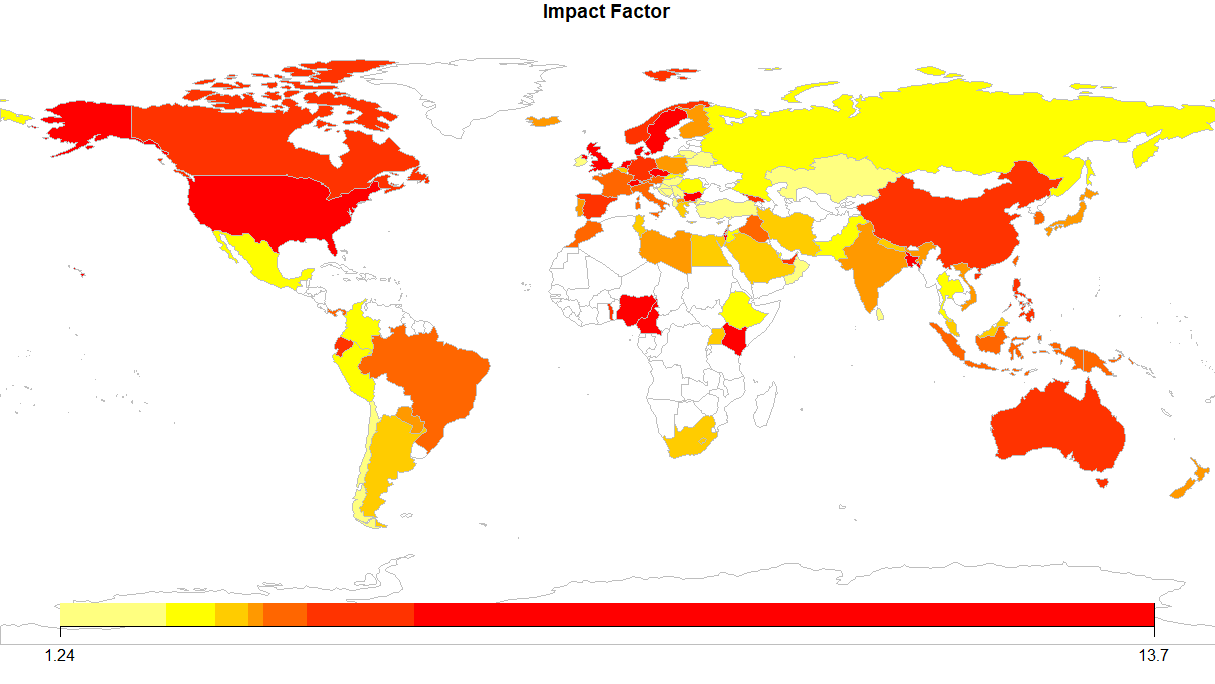


Supplementary Material 4. Mean number of Journal Impact Factor per country based on the corresponding author


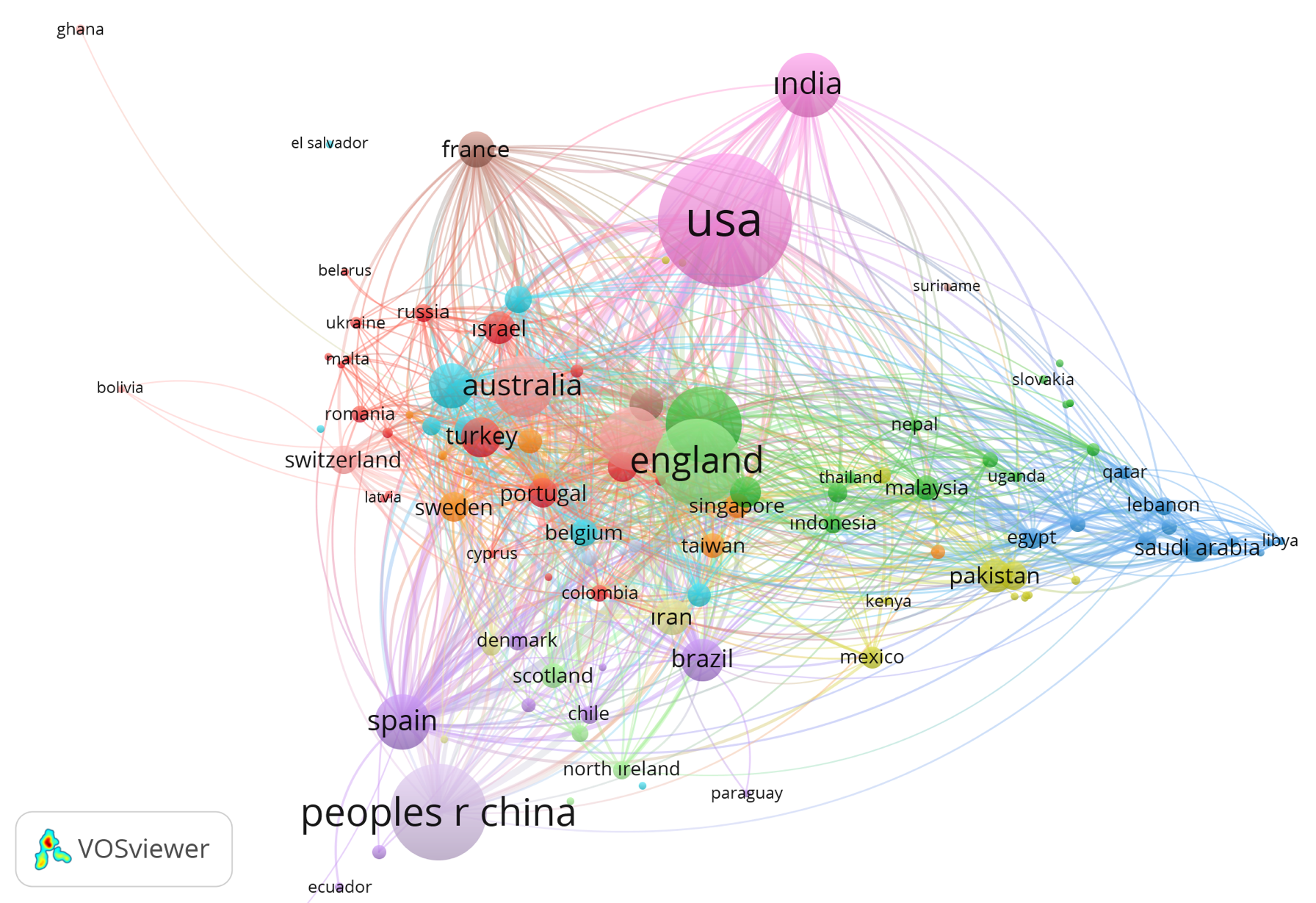


Supplementary Material 5. Inter-country collaborations in the VOSviewer software tool
