# Supplementary material for: Is everyone invited to the discussion table? A bibliometric analysis COVID-19-related mental health literature
Source: Glob Ment Health (Camb). 2022 Jul 29;9:366–74. doi: 10.1017/gmh.2022.37 (PMC9379265; doi:10.1017/gmh.2022.37)
Supplement: Supplementary file 1 [file S2054425122000371sup.zip › S2054425122000371sup002.docx]

Supplementary material 6. Description of the top 10 cited papers

| **No** | **Title** | **Country**  **of Origin** | **Citations** | **Journal** | **JIF** | **Index** | **Quartile Category** | **OA** |
| --- | --- | --- | --- | --- | --- | --- | --- | --- |
| 1 | Immediate Psychological Responses and Associated Factors during the Initial Stage of the 2019 Coronavirus Disease (COVID-19) Epidemic among the General Population in China | China | 1569 | International Journal of Environmental Research and Public Health | 3.390 | SSCI | Q1 | ✓ |
| 2 | Factors Associated with Mental Health Outcomes Among Health Care Workers Exposed to Coronavirus Disease 2019 | China | 1242 | JAMA Network Open | 8.483 | SCIE | Q1 | ✓ |
| 3 | Multidisciplinary research priorities for the COVID-19 pandemic: a call for action for mental health science | UK | 800 | Lancet Psychiatry | 27.083 | SCIE | Q1 | X |
| 4 | The psychological impact of the COVID-19 epidemic on college students in China | China | 577 | Psychiatry Research | 3.222 | SSCI | Q2 | X |
| 5 | Generalized anxiety disorder, depressive symptoms and sleep quality during COVID-19 outbreak in China: a web-based cross-sectional survey | China | 513 | Psychiatry Research | 3.222 | SSCI | Q2 | X |
| 6 | COVID-19 and mental health: A review of the existing literature | India | 505 | Asian Journal of Psychiatry | 3.543 | SCIE | Q2 | X |
| 7 | A longitudinal study on the mental health of general population during the COVID-19 epidemic in China | Singapore | 446 | Brain Behavior and Immunity | 7.217 | SCIE | Q1 | X |
| 8 | Knowledge, attitudes, and practices towards COVID-19 among Chinese residents during the rapid rise period of the COVID-19 outbreak: a quick online cross-sectional survey | China | 433 | International Journal of Biological Sciences | 6.580 | SCIE | Q1 | ✓ |
| 9 | Prevalence of depression, anxiety, and insomnia among healthcare workers during the COVID-19 pandemic: A systematic review and meta-analysis | UK | 358 | Brain Behavior and Immunity | 7.217 | SCIE | Q1 | X |
| 10 | Impact of COVID-19 pandemic on mental health in the general population: A systematic review | Canada | 344 | Journal of Affective Disorders | 4.830 | SSCI | Q1 | X |

SCIE: Science Citation Index Expanded™, SSCI: Social Sciences Citation Index™, ESCI: Emerging Sources Citation Index™, GDP: Gross Domestic Product, JIF: Journal Impact Factor (WoS Journal Citation Reports ™ 2020); OA: Open Access

Supplementary Material 7. Additional information about the top 10 cited papers

| **No** | **Corresponding Affiliation** | **Aim of the Study** | **Study Period** | **Study Type** | **Number of participants** |
| --- | --- | --- | --- | --- | --- |
| 1 | Institute of Cognitive Neuroscience | Determination of the prevalence of psychiatric symptoms and identification of the risk and protective factors contributing to psychological stress | January-February 2020 | Cross-sectional study | 1210 |
| 2 | Department of Psychiatry | To quantify the importance of symptoms of depression, anxiety, insomnia, and distress and analyze their potential risk factors to evaluate mental health outcomes among healthcare workers working with people with COVID-19 | January-February 2020 | Cross-sectional study | 1257 |
| 3 | Department of Psychology and Department of Clinical Neuroscience | Evaluation of the mental health science research priorities for the COVID-19 outbreak and ensure a strategy that may be adapted and integrated for the other countries’ research efforts | March-April 2020 | Position Paper | Not available |
| 4 | Department of Preventive Medicine | Evaluation of the mental situation of college students during the epidemic, to provide a theoretical basis for psychological interventions with college students and supply a basis for the announcement of national and governmental policies | Not available | Cross-sectional study | 7143 |
| 5 | Institute of Postgraduate Medical Education and Research | To summarize the current literature addressing mental health concerns related to the COVID-19 pandemic | Not available | Review | 28 articles |
| 6 | Department of Rehabilitation Medicine | Evaluation of the mental health burden of the Chinese public during the COVID-19 epidemic, and exploring the potential influence elements | February 2020 | Cross-sectional study | 7236 |
| 7 | Institute of Cognitive Neuroscience | Evaluation of the temporal psychological effects and adverse mental health situation during the beginning of the outbreak and peak of the COVID-19 epidemic and identity risk and protective factors for the Chinese population | January-March 2020 | Longitudinal study | 2271 |
| 8 | Department of Psychiatry | Examination of the knowledge, attitudes, and practices for COVID-19 of Chinese citizens in the time of the rapid rise period of the COVID-19 pandemic. | January-February 2020 | Cross-sectional | 6910 |
| 9 | Department of Psychiatry | Investigation of the emerging evidence of the effects of the COVID-19 pandemic on healthcare workers’ mental health and especially in association with the prevalence of insomnia, anxiety, and depression. | Until April 2020 | Systematic review and meta-analysis | 13 articles |
| 10 | Department of Pharmacology and Toxicology | To summarize the present literature that reported on the prevalence of symptoms of depression, anxiety, post-traumatic stress disorder, and different types of psychological distress in the population through the COVID-19 pandemic. Also, identification of the psychological distress factors. | Until May 2020 | Systematic review | 19 articles |
